# Trajectories of Change, Illness Understanding, and Parental Worries in Children and Adolescents Undergoing Internet-Delivered Cognitive-Behavioral Therapy for Functional Abdominal Pain Disorders: Protocol for a Single-Case Design and Explorative Pilot Study

**DOI:** 10.2196/58563

**Published:** 2025-01-07

**Authors:** Eva Skovslund Nielsen, Karen Kallesøe, Tine Bennedsen Gehrt, Ellen Bjerre-Nielsen, Maria Lalouni, Lisbeth Frostholm, Marianne Bonnert, Charlotte Ulrikka Rask

**Affiliations:** 1 Department of Child and Adolescent Psychiatry Aarhus University Hospital Aarhus N Denmark; 2 Department of Clinical Medicine Aarhus University Aarhus N Denmark; 3 Department of Research and Development Prehospital Emergency Medical Services, Central Denmark Region Aarhus N Denmark; 4 Division of Neuro Department of Clinical Neuroscience Karolinska Institutet Solna Sweden; 5 Center for Epidemiology and Community Medicine Health Care Services Stockholm County Stockholm Sweden; 6 Department of Functional Disorders and Psychosomatics Aarhus University Hospital Aarhus N Denmark; 7 Centre for Psychiatry Research, Department of Clinical Neuroscience Karolinska Institutet & Stockholm Health Care Services Stockholm Sweden

**Keywords:** functional abdominal pain disorders, abdominal pain, internet-based intervention, cognitive behavioral therapy, interoception, attentional bias, parental distress, single case study, children, adolescents, youth, study protocol, quality of life, treatment, medication, psychological treatment, psychology

## Abstract

**Background:**

Functional abdominal pain disorders (FAPDs) are common in young people and are characterized by persistent or recurrent abdominal symptoms without apparent structural or biochemical abnormalities. FAPDs are associated with diminished quality of life, school absence, increased health care use, and comorbid anxiety and depression. Exposure-based internet-delivered cognitive behavioral therapy (ICBT) has demonstrated efficacy in alleviating abdominal symptoms and improving quality of life. However, a deeper understanding of effect mechanisms and identification of possible additional treatment targets could refine treatment.

**Objective:**

This protocol paper aims to describe a study focusing on children and adolescents undergoing ICBT for FAPDs, aiming to further investigate the underlying mechanisms of effect.

**Methods:**

Children (8-12 years), adolescents (13-17 years) with FAPDs, and their respective parents will be included for 10 weeks for ICBT. First, detailed trajectories of effect are examined through a randomized single-case design study involving 6 children and 6 adolescents (substudy 1). Following this, an open-ended explorative pilot study with 30 children and 30 adolescents explores potential illness-related cognitive biases and interoceptive accuracy before and after treatment (substudy 2). Finally, spanning across these 2 substudies, including all parents from substudies 1 and 2, we will assess parental distress and illness worries before and after treatment, and how these factors impact the treatment adherence and outcomes of the child or adolescent (substudy 3).

**Results:**

Recruitment of participants began in June 2022 and is finalized for substudy 1 and ongoing for substudies 2 and 3. Recruitment is expected to be completed by January 2025, with final data collection during April 2025.

**Conclusions:**

The findings have the potential to contribute to the ongoing improvement of specialized psychological treatment for FAPDs in young people.

**Trial Registration:**

ClinicalTrials.gov NCT05237882; https://clinicaltrials.gov/study/NCT05237882; ClinicalTrials.gov NCT05486585; https://clinicaltrials.gov/study/NCT05486585; OSF Registries osf.io/c49k7; https://osf.io/c49k7

**International Registered Report Identifier (IRRID):**

DERR1-10.2196/58563

## Introduction

Functional abdominal pain disorders (FAPDs) affect up to 15% of children and adolescents and are characterized by recurrent or persistent abdominal pain and other debilitating gastrointestinal symptoms such as changes in defecation patterns and nausea [1,2]. The disorders are associated with reduced quality of life, high absence from school, and increased health care use [3]. Psychiatric comorbidities, especially anxiety and depression, are prevalent [4] and up to 40% persist in reporting abdominal symptoms into adulthood [5].

The pathophysiology of FAPDs is not fully understood but recent research suggests a biopsychosocial perspective where the complex interactions of physiological processes such as visceral hypersensitivity, psychological factors like emotional distress and anxiety, and social factors including family dynamics and environmental influences can contribute to the development and maintenance of gastrointestinal symptoms [6]. These factors combined are related to altered processing of sensory stimuli along the brain-gut axis with persistent or recurrent experience of abdominal pain and other gastrointestinal symptoms [7-10]. This altered processing can be understood within the framework of predictive processing, a theory suggesting that the brain consistently evaluates predictions regarding sensory inputs and discrepancies from these predictions [11]. Crucial factors contributing to the development of maladaptive predictive processes encompass cognitive biases marked by symptom-related fear and catastrophizing with an attentional bias toward pain or gastrointestinal stimuli [12-15]. This may be accompanied by the avoidance of situations expected to trigger symptoms [4,16-18] and changes in the interoceptive ability to sense, process, and interpret body signals [19]. Parental behaviors, such as solicitous responses, fearful communication about symptoms, and encouraging avoidance of situations that may provoke symptoms, can further influence the child’s perception of bodily stimuli [20-23].

In summary, understanding the pathophysiology of FAPDs involves navigating a complex system of various factors and processes. Consequently, treatment may encompass different targets, including both child-specific and contextual, that is, typically parental-specific factors.

In line with this, cognitive behavioral therapy (CBT) is the treatment supported by the strongest evidence of effect [24-26], with its main focus on restructuring potential child symptom-related maladaptive cognitions, emotions, and behaviors, and often with parental involvement. Swedish studies have documented the efficacy of internet-delivered cognitive behavioral therapy (ICBT) aimed at children and adolescents with FAPDs and their parents [27-29]. The ICBT focuses especially on exposure exercises and parental management of their child or adolescent’s symptoms. By enabling the child or adolescent to manage symptoms in previously avoided situations and thereby minimizing the gastrointestinal-specific fear, the treatment reduces the proposed visceral hypersensitivity, which over time leads to fewer abdominal symptoms.

Still, a proportion of the young patients do not improve from ICBT as the number needed to treat has been reported to be around 4, meaning approximately 4 patients must receive the treatment for 1 additional patient to experience adequate symptom relief [27,29]. Therefore, a deeper understanding of the mechanisms of change and the potential influence of additional modifiable factors on the treatment effect is required to improve treatment effects even more.

In the current study, we will evaluate translated versions of the Swedish ICBT for children and adolescents with FAPDs in a Danish context. The treatments target both children (aged 8-12 years) and adolescents (aged 13-17 years) and their parents, which provides a unique possibility to examine both child or adolescent and parental factors before and after treatment. The aims of the study are to (1) investigate the detailed trajectory of the effect of ICBT in children and adolescents with FAPDs, (2) explore potential illness-related cognitive biases and interoceptive accuracy in children and adolescents with FAPDs compared with healthy controls, and if these factors are changed after ICBT, and (3) explore if parental distress, illness worries, and behaviors may impact the child or adolescent’s treatment adherence and outcome.

This protocol article is reported following the SPIRIT (Standard Protocol Items Recommendations for Interventional Trials) guidelines [30].

## Methods

### Study Design

The study includes 2 recruitment phases. The first phase concerns a single case experimental design (SCED) study (substudy 1) with a total of 12 patients (6 children and 6 adolescents, respectively). The second phase concerns an open pilot study on cognitive biases and interoceptive accuracy (substudy 2) with a total of 60 patients (30 children and 30 adolescents, respectively).

**Figure 1:**
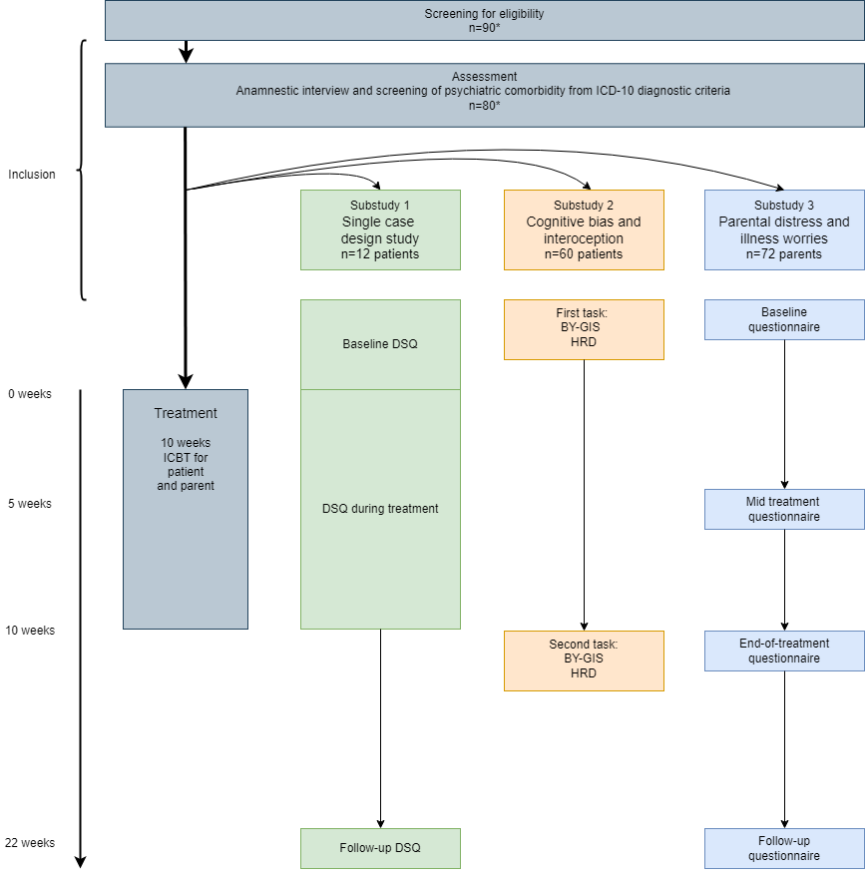
Overall study design. BY-GIS: biases in youth toward gastrointestinal-related stimuli; DSQ: daily short questionnaire; HRD: heart rate discrimination; ICBT: internet-delivered cognitive behavioral therapy; ICD-10: International Statistical Classification of Diseases, Tenth Revision.

Consequently, a total of 72 children and adolescents, along with a corresponding number of parents, will undergo ICBT for FAPDs throughout the entire study. Parental distress and illness worries will be assessed before, during, and after the treatment in all included parents (substudy 3; Figure 1).

### Study Setting and Recruitment

Children and adolescents diagnosed with FAPD will be referred to the project from pediatric departments located at 4 hospitals (1 university hospital and 3 regional hospitals, respectively) as well as from private pediatric practitioners in the Central Denmark Region. Due to a slow referral rate of adolescents, we have been granted permission from The Central Denmark Region Committees on Health Research Ethics to expand the inclusion area to include additional regions (the North Denmark Region and the Region of Southern Denmark). We will maintain continuous communication with referring pediatricians to ensure sufficient participant enrollment. This will include in-person meetings, emails, and newsletters.

### Eligibility Criteria

Eligible participants will be invited for an assessment interview at the Department of Child and Adolescent Psychiatry, Aarhus University Hospital Psychiatry. The assessment will include psychiatric comorbidities, such as neurodevelopmental disorders, mood disorders, and suicidal risk, systematically evaluated according to the *ICD-10* (*International Statistical Classification of Diseases, Tenth Revision*) diagnostic criteria. Assessments will be performed by medical doctors who are either trainees or specialists in child and adolescent psychiatry.

#### Inclusion Criteria

First, age 8-17 years. Second, a primary diagnosis according to the ROME-IV criteria of one of the FAPD subtypes—irritable bowel syndrome (IBS) or functional abdominal pain not otherwise specified (FAP-NOS) documented by the child’s regular pediatric physician [31]. The somatic evaluation includes recommended routine medical investigations, that is, growth, fecal calprotectin, and blood samples (TSH [thyroid stimulating hormone], total IgA [immunoglobulin A], IgA-tissue transglutaminase, complete blood count, C-reactive protein analysis, and liver enzymes). Third, stable dosage of regular FAPD-related medication such as laxatives, antidiarrheal medicines, or psychopharmacological medication during the past month.

#### Exclusion Criteria

First, another medical or psychiatric disorder that better explains the symptoms. Second, severe child psychiatric or social problems (eg, high level of suicidal ideation, school absence of more than 40% during the past month, or ongoing substance abuse). Third, ongoing psychological treatment. Fourth, insufficient language or computer skills (patients and parents). Fifth, severe family problems (eg, child maltreatment, parental substance abuse or severe psychiatric illness, and custody fight).

### Intervention

#### Translation Procedure

For this study, the Swedish ICBT programs were translated into Danish. The translation procedure was stepwise; first, a direct translation was performed by native Danish speakers familiar with the Swedish language; second, a mutual discussion of the Danish translation took place within the research group, including a discussion of problematic sentences or wordings; third, remaining issues were discussed with the Swedish authors, and a final consensus on the Danish translation was reached. This translation was reviewed by both a Danish language expert and a clinician with experience with the patient group to make further refinements. Finally, in the last step, the revised translation was reviewed again by the Danish research group for final adjustments.

#### Design and Content of Treatment

The 4 distinct ICBT programs used in this study are targeted children (8-12 years), adolescents (13-17 years), and the parents of each group, respectively. The treatment lasts for 10 weeks and consists of 10 modules for children, adolescents, and parents of children (with a new module every week) and 5 modules for parents of adolescents (with a new module every other week). An overview of treatment content can be seen in Table 1. The programs include sections with videos, exercises, and quizzes designed to actively engage the child or adolescent in the treatment. Psychoeducation regarding FAPDs, detection of avoidance behavior, a brief mindfulness exercise called “SOL (Stop, observe, let go)” and graded repeated exposure exercises comprise some of the central components covered in the programs for both children and adolescents. The first modules of treatment introduce the above-mentioned concepts whereas later modules focus on repeated exposures. The purpose of “SOL” is Stopping, Observing how the stomach feels, and Letting go of the focus on the stomach and continuing with the activity. The parental programs emphasize supporting their child or adolescent’s exposure, decreasing attention to their child or adolescent’s abdominal symptoms, and prioritizing shared positive activities. Each module ends with planning of the homework assignments which are evaluated first thing in the next module.

**Table 1:** Overview of the content of treatment for each module.

Module	Children (8-12 years)	Parents	Adolescents (13-17 years)	Parents
1	Introduction to the treatment format Psychoeducation on FAPDs^a^ (video) Mapping “stomach behaviors” (control, avoidance, safety) Setting treatment goals Homework: Self-monitoring	Introduction to the treatment format Positive attention Focus shift Mapping common parental behaviors Handling personal frustrations Homework: Focus shift, breaks, involving peers	Introduction to the treatment format Psychoeducation on FAPDs (video) Setting treatment goals Homework: self-monitoring	Introduction to the treatment format Focus on positive attention and shared moments Mapping common parental behaviors Homework: Shared positive moments
2	Psychoeducation on impact of thoughts and “SOL”^b^ (video) Constructing an exposure hierarchy. Homework: Utilize “SOL” in everyday situations.	Golden moments Board game with rewards for exposure exercises. Homework: Golden moments, focus shift, planning rewards”	Mapping “stomach behaviors” (control, avoidance, safety) Behavior analysis Homework: Behavioral experiment (avoiding “stomach behaviors”)	
3	Behavior analysis Psychoeducation on exposure exercises (video) Homework: Exposure exercises.	Supporting child’s exposure exercises Managing school absences Homework: board game rewards, golden moments, focus shift	Psychoeducation on impact of thoughts and “SOL” (video) Toilet habits (frequent visits, urgency) Homework: “SOL” and new toilet habits	Psychoeducation on FAPDs (video) Acknowledging and shifting focus to reduce symptom focus Homework: Shared positive moments, letting go of parental behaviors, focusing on alternative adolescent behaviors
4	Toilet Habits (frequent visits, urgency) Behavior analysis Homework: Exposure exercises, new toilet habits, “SOL”	Parental stress and recreational activities Homework: recreation, golden moments, board game rewards, focus shift	Psychoeducation on exposure exercises (video) Behavior analyses Construct exposure hierarchy Homework: “SOL,” toilet habits, finish exposure hierarchy	
5	Repetition Behavior Analysis Homework: Exposure exercises, toilet habits, eliminating safety behaviors, “SOL”	Repetition Mapping challenges Homework: recreation, golden moments, board game rewards, focus shift	Exposure exercises Anticipatory anxiety Emotional versus behavioral goals Homework: Exposure exercises, “SOL,” toilet habits.	Encourage and support the adolescent’s exposure Psychoeducation on exposure exercises (video) Homework: Shared positive moments, support and encouragement, support adolescent’s exposure
6	Positive analysis of new behaviors Advancing exposure difficulty Homework: Advancing exposure exercises, toilet habits, “SOL”	Problem-solving with child Homework: recreation, golden moments, board game rewards, focus shift, problem-solving	Further details on exposure and rewards Homework: Exposure exercises, “SOL,” and toilet habits	
7	Positive behavior analyses Evaluation of Treatment Goals (from Module 1) Homework: Exposure exercises, toilet habits, “SOL”	Parental behavior analyses in child interaction Homework: recreational, golden moments, board game rewards, focus shift	Level up exposure exercises Homework: Exposures exercises, “SOL,” toilet habits	Managing personal frustrations Engaging in recreational activities Listening effectively Homework: Shared positive moments, support and encouragement, support adolescent’s exposure, active listening, parental recreation
8	Positive behavior analyses Advancing exposures to a “Super Hard Day” Homework: Exposure exercises, the “Super Hard Day,” toilet habits, “SOL”	Review of progress Reward for efforts Homework: recreation, golden moments, board game rewards, focus shift, parental reward	Emphasis on more challenging exposures Homework: Challenging exposure exercises, “SOL,” toilet habits	
9	Treatment repetition using quizzes Progress evaluation Homework: Final exposure exercises, toilet habits, “SOL”	Progress evaluation Challenge evaluation Homework: recreation, golden moments, board game rewards, focus shift.	Focus on how to further challenge oneself. Homework: Exposure exercises	Review of treatment Parental behavior evaluation (comparison with module 1) Future training and relapse prevention plan
10	Treatment goal and exposure hierarchy evaluation “Stomach behavior” evaluation (comparison with module 1) Future training and relapse prevention plan	Parental behavior evaluation (comparison with module 1) Future training and relapse prevention plan	Treatment goal and exposure hierarchy evaluation “Stomach behavior” evaluation (comparison with module 1) Future training and relapse prevention plan	

^a^FAPDs: Functional abdominal pain disorders.

^b^SOL: Stop, observe, let go.

#### Modifications and Web Page

The Danish versions of the ICBT programs were pretested in 2 children and 2 adolescents with biweekly feedback from the patients and parents on overall experience and content (telephone interviews performed by a research assistant). Minor modifications were performed, mainly in the adolescent program where the IBS treatment was adjusted to include IBS and FAP-NOS. Furthermore, the mindfulness exercise “SOL” was integrated in the adolescent program to further support the process of labeling thoughts and sensations.

**Figure 2:**
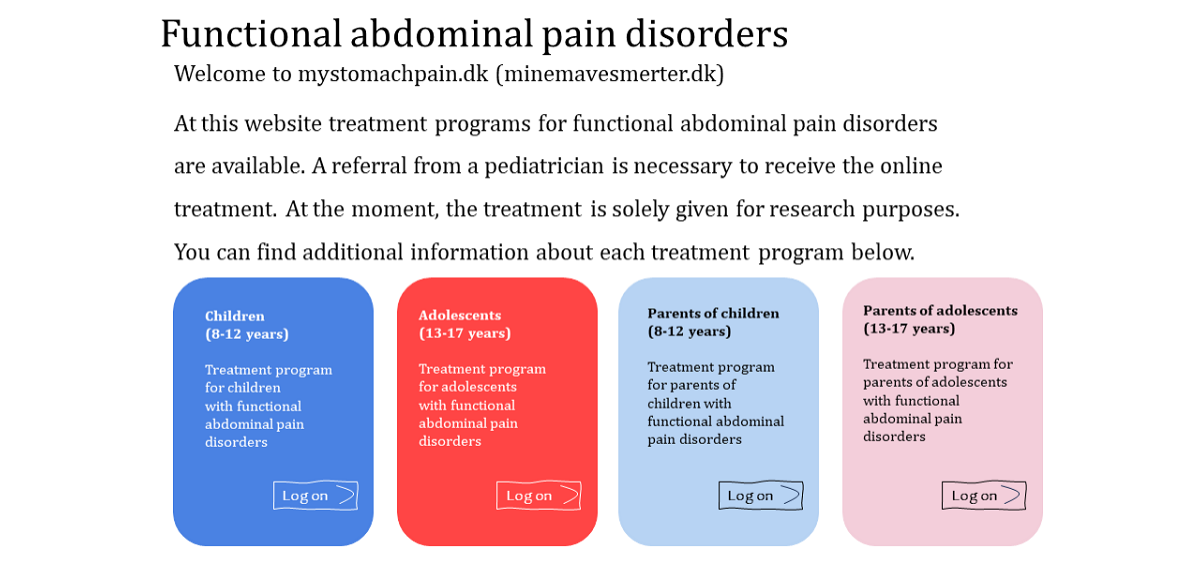
Screenshot of the start page for the Danish treatment programs (all text is translated from Danish to English).

All treatment elements are delivered through a web page specifically designed for this study (Figure 2). Each family will choose 1 primary parent to participate in the treatment at the assessment. All 4 treatment programs are supported by a therapist who provides written feedback after each module. The affiliated therapists will be psychologists and medical doctors with knowledge of CBT. They will receive weekly supervision from Danish specialists in CBT and further supervision from the Swedish research team, who developed and tested the original programs.

### Measures

#### Measures in the Single Case Design Study (Substudy 1)

Children and adolescents will answer a daily short questionnaire (DSQ) throughout a pretreatment baseline period, randomized to last between 5 and 15 days, throughout the 10 weeks of treatment, and for a final 7-day follow-up period 3 months after treatment. The DSQ is an 8-item questionnaire, designed in line with the SCRIBE (Single-Case Reporting guideline In BEhavioural interventions) guidelines [32], addressing specific targets of the intervention, with items from different validated questionnaires for children and adolescents [32,33]. The full DSQ questionnaire is shown in Textbox 1. The primary outcome in the SCED study is the level of gastrointestinal symptoms assessed using 2 items from the Pediatric Quality of Life Inventory (PedsQL) Gastrointestinal Symptoms Questionnaire [34]. Secondary outcomes include illness worries, avoidance behavior, and level of pain acceptance as measured by the Visceral Sensitivity Index (VSI), irritable bowel syndrome behavioral response questionnaire (IBS-BRQ), and the Chronic Pain Acceptance Questionnaire – Adolescent Short Form (CPAQ-A8), respectively [35-37]. The items have been modified into questions concerning the last 24 hours and are rated on a 0-10 scale (“not at all” 0, “all the time” 10).

**Daily short questionnaire (DSQ).**

During the last 24 hours, on a scale from 0 (not at all) to 10 (all the time)

1) Did your stomach hurt?

2) Did you feel discomfort in your stomach?

3) Did you worry about the problems in your belly?

4) Were you frightened when you felt discomfort in your belly?

5) Did you avoid going out in case you had belly problems?

6) Did you spent more time on the toilet than you ideally would like?

7) Did you do things that are important and things that are fun even though you have problems in your belly?

8) Has the most important thing been to keep your symptoms under control whenever you did something?

#### Measures on Cognitive Bias and Interoception (Substudy 2)

Cognitive biases will be assessed before and after treatment using a newly developed experimental design with a computerized web-based task: biases in youth toward gastrointestinal-related stimuli (BY-GIS) [38]. The BY-GIS task consists of a combination of a word task, which is a modified version of the Health Norms Sorting Task [39-41] and a picture task inspired by Gehrt et al [42]. The BY-GIS task is adapted to be appropriate for children and adolescents, featuring gastrointestinal-related stimuli (ie, words related to abdominal symptoms and pictures of food, leisure, and school situations). The BY-GIS task has 3 phases—encoding, free recall, and recognition. In the encoding phase, participants are asked to rate words and pictures presented to them. In the free recall phase, participants are instructed to recall as many words and pictures from the encoding phase as possible, and in the recognition phase, they are presented with words and pictures from the encoding phase along with new words and mirror images of the original pictures. They are then asked to mark which items have been presented to them before. The development, test, and full procedure of the BY-GIS task are described by Bjerre-Nielsen et al [38].

Interoception will be investigated before and after treatment using a psychophysical measure of cardiac interoception, that is, the heart rate discrimination (HRD) task, developed by Legrand et al [43]. During the HRD task, participants have their heart rate monitored using a pulse oximeter while they are presented with auditory and visual stimuli from a computer and answer questions about their sensations of heart rate and subjective confidence in their answers. Thereby, the interoceptive performance (psychometric threshold, slope, and reaction time) and the metacognitive performance (confidence) can be measured. In collaboration with researchers from the Center of Functionally Integrative Neuroscience at Aarhus University, the task was adapted for a younger age group and pretested in 3 children and adolescents. Adaptations consisted of shortening the test by reducing the number of repetitions of trials, as well as replacing written instructions about the conduction of the test with direct oral guidance from a research assistant.

For both studies in substudy 2, we will perform parallel studies on healthy controls. The BY-GIS task was already tested in 96 healthy controls [38], and the interoception test setup will be performed in 60 healthy controls (all aged 8-17 years).

#### Measures of Parental Factors and Child Treatment Effects (Substudy 3)

To assess how parental distress, illness worry, and behavior impact the child’s overall treatment effects in various domains and treatment adherence, all participating parents and children or adolescents in the ICBT programs will answer extensive questionnaires before assessment (T1: baseline), halfway through treatment (T2: mid-treatment), at end of treatment (T3: end of treatment), and 3 months after the end of treatment (T4: follow-up; Figure 1). Furthermore, the number of completed treatment modules will be obtained from the treatment web page after the end of treatment. The specific measures to be used are shown in Table 2 and described subsequently.

**Table 2:** Overview of questionnaires in substudy 3.

Outcome			Instrument	Time points and respondents^a^			
				T1	T2	T3	T4
**Parental factors**							
	Parental distress		SCL-8^b^ [44,45]	P	P	P	P
	Parental illness worries		HAPYS^c^ [46,47]	P	P	P	P
	Parental illness behavior		ARCS^d,e^ [48-50]	P	P	P	P
**Child treatment effects**							
	**Main measures**						
		Gastrointestinal symptoms	PedsQL Gastro^f^ [34]	C, A, P	C, A, P	C, A, P	C, A, P
		Pain intensity	Faces Pain Rating Scale – revised [51,52]	C, A, P	C, A, P	C, A, P	C, A, P
	**Additional measures**						
		Quality of life	PedsQL^g^ [53]	C, A, P	C, A, P	C, A, P	C, A, P
		Overall symptom load	CSSI^h^ [54]	C, A, P		C, A, P	C, A, P
		Depressive symptoms	MFQS^i^ [55,56]	C, A		C, A	C, A
		General anxiety	SCAS-S^j^ [57,58]	C, A, P		C, A, P	C, A, P
		Gastrointestinal anxiety	VSI^k^-short [35,59]	C, A	C, A	C, A	C, A
		Avoidance and control behavior	IBS-BRQ-C^l^ [36,60]	C, A	C, A	C, A	C, A
		Illness perception	B-IPQ^m^ [61,62]	C, A, P	C, A, P	C, A, P	C, A, P
		Illness worries	CIAS^n,o^ [63]	C, A	C, A	C, A	C, A
		Pain acceptance	CPAQ-A8^p,q^ [37]	C, A	C, A	C, A	C, A
**Other measures**							
	Adverse events		Yes or no, and open question			A, P	
	Treatment satisfaction		ESQ^r^ [64]			C, A, P	
	School absence		Hours past month	A, P	A, P	A, P	A, P
	Work absence		Days past month	P	P	P	P

^a^Time points; T1: baseline, T2: mid-treatment, T3: end-of-treatment, T4: follow-up; Respondents: C: child, A: adolescent, P: parent.

^b^SCL-8: Symptom Check List-8.

^c^HAPYS: The Health Anxiety by Proxy Scale.

^d^ARCS: Adult Responses to Children’s Symptoms.

^e^The monitor and protect subscales.

^f^PedsQL Gastro: Pediatric Quality of Life Inventory – Gastrointestinal symptoms.

^g^PedsQL: Pediatric Quality of Life Inventory.

^h^CSSI: Children’s Somatic Symptom Inventory.

^i^MFQS: Mood and Feelings Questionnaire Short.

^j^SCAS-S: Spence Children Anxiety Scale – Short.

^k^VSI: Visceral Sensitivity Index.

^l^IBS-BRQ-C: irritable bowel syndrome behavioral response questionnaire – Child-adapted short version.

^m^B-IPQ: Brief Illness Perception Questionnaire.

^n^CIAS: Childhood Illness Attitude Scale.

^o^The fear factor subscale.

^p^CPAQ-A8: Chronic Pain Acceptance Questionnaire – Adolescent Short Form.

^q^One question from the pain willingness factor, one from activity engagement.

^r^ESQ: Modified Experience of Service Questionnaire.

#### Parental Factors

Parental distress will be assessed by the Symptom Checklist-8, an 8-item subscale from the Symptom Checklist Revised-90, screening for emotional symptoms. Items are rated on a 5-point scale from 0 “not at all” to 4 “a whole lot,” with a total range from 0 to 32. Higher scores indicate more emotional distress [44,45].

Parental illness worries will be assessed by the Health Anxiety by Proxy Scale (HAPYS), a newly developed questionnaire assessing parents’ worries about their child’s health [46]. The questionnaire covers 26 items about health anxiety by proxy, rated on a 5-point scale (from 0 “not at all” or “never” to 4 “a whole lot” or “most of the time”), with a sum score range from 0 to 104. In addition, there are 6 items about the impact of the worries rated on a 4-point scale (from 0 “no” to 3 “yes, severely”), with a sum score range from 0 to 18. Higher scores indicate a high level of health anxiety by proxy and the impact of the anxiety, respectively. HAPYS is a valid measure of health anxiety by proxy with good psychometric properties, including high internal reliability and known-groups validity [47].

Parental illness behavior will be measured by the Adult Responses to Children’s Symptoms, which is originally a 29-item questionnaire [48]. In this study, 2 subscales (monitor and protect subscales) comprising 15 items will be used. Items are rated on a 5-point scale from 0 “never” to 4 “always” with a score range from 0 to 60. Higher scores indicate a higher degree of monitoring and protective responses [49,50].

#### Child or Adolescent Treatment Effects—Main Measures

Gastrointestinal symptoms will be assessed by PedsQL gastrointestinal symptoms [34]. This is a 9-item questionnaire assessing gastrointestinal symptoms related to functional or organic gastrointestinal diseases, during the past month, on a 5-point scale from 0 “never” to 4 “almost always.” Scores are reversed and transformed into a 0-100 scale, with high scores indicating greater quality of life. Pain intensity will be assessed by the Faces Pain Rating Scale – revised (FPS-R) [51]. The FPS-R score includes 6 pictures of faces, each showing an increasing amount of pain and scored on a 2-step scale between “no pain” (0) and “worst pain” (10). The scale is validated in children and adolescents and found to be sensitive to change [52].

#### Child or Adolescent Treatment Effects—Additional Measures

Quality of life will be measured by the 23-item PedsQL [53], which is a widely used questionnaire to assess quality of life during the past month in children and adolescents. Items are scored on a 5-point scale from 0 “never” to 4 “almost always.” Scores are reversed and transformed into sum scores between 0 and 100, with high scores indicating higher quality of life.

Overall symptom load will be assessed by the Children’s Somatic Symptom Inventory. It measures somatic symptoms during the last 2 weeks using 24 items, rated on a 5-point scale from 0 “not at all” to 4 “a whole lot,” with a range in scores from 0 to 96. High scores indicate a high symptom load. The questionnaire is found to be reliable and psychometrically sound for children and adolescents with abdominal pain disorders [54].

Depressive symptoms will be measured using the Mood and Feelings Questionnaire Short. It comprises 13 items rated on a 3-point scale from 0 “not true” to 2 “true,” with a range from 0 to 26, where higher scores indicate more depressive symptoms. The questionnaire is found reliable for evaluating depressive symptoms in both clinical and community populations of children and adolescents [55,56].

Anxiety levels will be assessed with 2 different questionnaires—the Spence Children Anxiety Scale Short (SCAS-S) [57,58] for general anxiety, and the Visceral Sensitivity Index-Children (VSI-C) [35,59] for gastrointestinal-specific anxiety. SCAS-S contains 19 items which are answered on a 4-point scale from 0 “never” to 3 “always,” with a total range from 0 to 57, where higher scores indicate higher anxiety levels. The questionnaire is designed for use in children and adolescents, and the short version is validated for screening of anxiety levels in children and adolescents [58]. The VSI-C is a shortened child-adapted version of the VSI for adults [35], with 7 items answered on a 6-point scale from 0 “strongly disagree” to 5 “strongly agree,” with a range from 0 to 42, where higher scores indicate more GI-anxiety. It has been found reliable and valid in children and adolescents with FAPDs [60].

Avoidance and control behavior will be assessed by the IBS-BRQ–Child-adapted short version with 11 items, which is an adapted version of the IBS-BRQ for adults [36]. Items are rated on a 7-point scale from 0 “never” to 6 “always,” with scores ranging from 0 to 66, where higher scores indicate more avoidance. It has been validated for children and adolescents with FAPDs [60].

Illness perception will be assessed by the Brief Illness Perception Questionnaire [61,62], which is a 9-item questionnaire where 8 items are answered on an 11-point scale, and 1 item is an open-ended question. The total score range is 0-80, with higher scores indicating more severe illness perception.

Illness worries will be assessed by the Childhood Illness Attitude Scale with 11 items from the fear factor [63]. The questionnaire is developed and validated for children and adolescents. Items are answered on a 3-point scale from 0 “never” to 2 “most of the time,” with a total score range from 0 to 22, and higher scores indicate more illness worries.

Pain acceptance will be assessed by 2 items from the CPAQ-A8 [37]. One regarding pain willingness and one regarding activity engagement. They are both answered on a 5-point scale from 0 “never true” to 4 “always true,” the item on pain willingness is reversed scored, and higher scores then indicate higher pain acceptance.

#### Other Measures

Parents will provide information about school absence (hours last month) for children (aged 8-12 years) and about their work absence (days past month). Adolescents will provide information about their own school absence (hours last month).

Adverse events will be assessed by adolescents and parents of children using a binary (yes or no) question and an open-ended question to describe the potential event in detail.

Treatment satisfaction will be assessed by the Modified Experience of Service Questionnaire [64], which is a 13-item questionnaire with 10 items scored from “not true” to “certainly true” and 3 free-text items. The total score range is 0-20 with higher scores indicating a better experience with the treatment.

### Sample Sizes

According to research design standards [65], a minimum of 3 replications are recommended in a SCED study (substudy 1). However, to achieve sufficient power for randomization tests, a higher number of potential randomizations is recommended [66]. The number of randomizations is influenced by both the number of participants and the number of possible starting points; hence, guided by Levin et al [67], we chose 6 participants in each group.

For substudy 2, we based our power analysis on potential differences in cognitive bias and interoception between cases and healthy controls on a previous similar study on cognitive bias in children and adolescents [40] and studies on interoceptive accuracy in adults with functional disorders [68-69]. Based on these study findings, calculations show that in order to detect a minimum effect size of 0.5 (Cohen *d*), 60 patients and 100 healthy controls are required for the cognitive bias outcome, and a minimum of 51 patients and 51 healthy controls for the interoception outcome to achieve 80% power at an α level of .05. The subsequent analysis of the possible impact of treatment on these 2 factors is more explorative and therefore no a priori power calculation is provided. The same concerns the last study on parental factors (substudy 3) as possible effect moderators. However, here the sample size is the largest possible, comprised of parents from substudies 1 and 2.

### Data Collection and Management

The DSQ, the BY-GIS task, and questionnaires are set up in REDCap (Research Electronic data capture; Vanderbilt University) [70], hosted by Aarhus University, and distributed through SMS text message or email. REDCap is a secure, web-based software platform designed to facilitate data capture for research studies.

For the DSQ (substudy 1), a text message reminder will automatically be sent through REDCap 2 hours after the initial SMS text message. If the DSQ is not completed 2 days in a row, a research assistant will contact the family by phone. For the BY-GIS task (substudy 2) and the extensive questionnaire (substudy 3), REDCap will generate automatic email reminders 24, 48, and 72 hours after the initial email if they are not completed. If there is no response after 7 days, a research assistant will contact the family by phone.

The interoception task in substudy 2 will take place at the Center of Functionally Integrative Neuroscience at Aarhus University Hospital in a behavioral testing room, and data will be stored in REDCap.

### Data Analyses

In substudy 1, visual inspection analyses will be applied to demonstrate the effect of the intervention on all outcomes [71]. In addition, within-case effect size measurements will be calculated using Tau-U [72] and between-case effect sizes will be determined using hierarchical linear regression models to account for the serial dependency of data [73]. A randomization test will be applied to test differences in means for the baseline versus treatment, and baseline versus follow-up periods, respectively [74].

In substudy 2, participants’ descriptions reported in the recall phase of the BY-GIS task will be coded to their corresponding word or picture and category presented in the encoding phase (eg, picture-description “children and cake” coded as the birthday picture within the fun category). Coding for the first 20% of participants will be conducted by 2 independent raters (ESN and EB-N). If the consensus rating exceeds 90%, 1 rater will code the remaining cases independently. A third and experienced rater (TBG) may be consulted to resolve potential disagreements. Baseline data will be analyzed descriptively and compared with (1) data from a healthy control group who performed the same experimental test [38], and (2) end-of-treatment data, exploring potential differences in the outcomes from the encoding, recall, and recognition phases using *t* tests or nonparametric tests, depending on data variability in the samples.

Baseline measures of interoceptive performance and metacognitive measures will be analyzed descriptively and compared with (1) measures from a healthy control group and (2) end-of-treatment measures, using *t* tests or nonparametric tests, depending on data variability in the samples.

In substudy 3, the potential moderation of parental factors on treatment adherence (number of completed modules) and main measure (gastrointestinal symptoms) for children or adolescents will be investigated using negative binomial regression and linear mixed models, respectively, adjusting for baseline parental distress, illness worries, and illness behavior, respectively.

In addition, the potential effect of treatment on these parental factors and all other child or adolescent measures will be analyzed using linear mixed regression models.

An experienced statistician will provide statistical support for all analyses.

### Ethical Considerations

The study will be conducted according to the guidelines of the Declaration of Helsinki. The study was approved by The Central Denmark Region Committees on Health Research Ethics (record 1-10-72-277-21, 1-10-72-80-22, and 1-10-72-142-22).

Written informed consent will be obtained from parents and adolescents (≥15 years) whereas children and adolescents (<15 years) will only give oral consent.

Children and adolescents will be compensated with a gift card (value 150 DKK [US $21.13]) for the first time participating in the interoception study. No other compensation will be provided.

## Results

Recruitment of participants began in June 2022. Substudy 1 is finalized and expected to be published in 2025. Recruitment for substudies 2 and 3 is ongoing. By November 2024, 45 children and 24 adolescents have been included. Due to a skewed distribution in referrals, with a predominance of referrals on children, we continue to include children and adolescents until we have reached 30 adolescents. Inclusion is expected to be completed by January 2025 and data collection in March 2025.

## Discussion

### Principal Findings

With this study, we aim to explore previously sparsely investigated areas of ICBT for children and adolescents with FAPDs related to both child or adolescent and parental factors. This includes examining the detailed trajectory of effect, the presence of and potential changes in cognitive biases, and interoceptive inaccuracy after treatment, as well as investigating the potential role of parental distress, illness worries, and behavior as effect moderators.

Previous studies have demonstrated the effectiveness of Swedish ICBT programs in reducing abdominal symptoms and improving the quality of life in children and adolescents with FAPDs. Mediation analyses have shown that positive changes in symptom-specific avoidance behavior and fear mediate the treatment effects [17,18,27,29]. However, the detailed trajectory of treatment effects—specifically when and how possible changes occur during treatment for individual patients—remains unknown, and this will be investigated in substudy 1. This research could provide new insights into the mechanisms of change underlying treatment effects and could be clinically valuable for both therapists and patients.

To the best of our knowledge, this study is one of the first to assess cognitive biases and the ability to sense and interpret signals from the body (ie, interoception) in this patient group in comparison with healthy controls as well as to explore potential changes in these factors after treatment with CBT. Both factors are suggested to play a vital role in the predictive processing model, explaining the pathophysiology of FAPDs [11]. However, so far, they have mainly been studied in adults with various functional disorders [40,68-69,75-78]. By uncovering more knowledge about these factors, the results can potentially be used to further refine already established treatments. For example, this could involve adding biofeedback to train interoceptive accuracy or enhancing exposure exercises addressing specific cognitive biases that lead to fearful responses to gastro-related sensations and avoidance behavior.

Parental worries and anxious behaviors toward the child or adolescent’s symptoms can impact the overall well-being of the child or adolescent. These factors are suggested to be part of the complex pathophysiology of FAPDs [9,20,21,48] and are therefore important to investigate further. Parental emotional distress has been found to negatively moderate the effect of ICBT for adolescents with chronic pain [79,80], whereby high levels of parental emotional distress lead to less improvement in disability for the adolescent. However, it is currently unknown how the level of parental distress impacts the effects of the ICBT programs for young patients with FAPDs [29,81]. A specific type of parental emotional distress is health anxiety by proxy, which is a recently described clinical phenomenon characterized by parents’ excessive and distressing worries and rumination about their child’s health, often involving fears of serious diseases being overlooked by medical professionals [82,83]. This fear may lead to frequent medical visits and investigations of the child [84]. In this study, we will use the newly developed HAPYS questionnaire [46,47] to assess parental health anxiety by proxy. To our knowledge, this is the first systematic examination of this specific type of parental emotional distress in parents of children and adolescents with FAPDs. Such type of parental anxiety may especially lead to symptom-related protective and monitoring behaviors, which are suggested to play a crucial role in the development, maintenance, and even aggravation of the child’s FAPD and related health care visits [48-50]. The Swedish ICBT has been shown to effectively reduce such maladaptive parental behavior in both children and adolescents [28,29]. Still, the impact of parental emotional distress, particularly illness worries and related behavioral responses, on the effect of ICBT in children is less well-investigated [85] and no studies have yet been conducted specifically in relation to adolescents. This is why, in this study, we will explore parental distress, illness worries, and behaviors before and after ICBT and examine how these factors impact treatment adherence and treatment effects in both children and adolescents. By uncovering more knowledge about these parental factors, we aim to gain a better understanding of family dynamics and identify potentially important parental characteristics to focus on during treatment, thereby further improving its effectiveness.

The study has some limitations. In substudy 1, the generalizability of SCED study results has traditionally been questioned due to the low number of participants. However, our study adheres to guidelines that include multiple data points across different phases, randomization of the length of the baseline phase, and replication across several participants, thereby optimizing generalizability. Furthermore, participants are expected to answer daily questions for an extended period, which could induce some fatigue in responding and potentially affect reliability. Still, the inclusion of several close data points increases precision in analyses and helps buffer against occasional data loss. In addition, the setup was tested on 4 prestudy patients, who reported that the daily questionnaire was easy and quick to answer.

In substudy 2, we use the BY-GIS task, which is newly developed and has not been previously tested in patients. However, the task is developed based on well-known paradigms and previous tests, and it has already been tested in a sample of 96 healthy age-matched controls with good usability [38]. The interoception HRD measures participants’ ability to sense their pulse, which is not directly related to gut sensations but falls within the broader perspective of interoception. However, studies in adults suggest altered interoceptive accuracy across different diagnoses of functional disorders and chronic pain, which indicates that there may be a general hypersensitivity to inner sensations in this patient group as well [86]. Since this study is one of the first to investigate interoceptive accuracy in children and adolescents with FAPDs, we believe it is still valuable to use this well-known and widely used interoception measure.

Substudy 3 is an open pilot trial without a control group for comparison which means conclusions regarding the clinical effect of the treatment should be interpreted with caution. However, the substudy is explorative and primarily aims to investigate new and additional modifiable treatment targets on a parental level.

### Conclusion

In conclusion, this study is expected to provide new and important information about the process of the effect of ICBT, increase our understanding of modifiable treatment targets within the framework of predictive processing in children and adolescents with FAPDs, and shed light on parental factors that are important for the child’s treatment adherence and effect. Overall, this research can guide the further development of even more effective psychological treatments for one of the most prevalent chronic pain disorders in children and adolescents.

## Abbreviations

BY-GIS: biases in youth toward gastrointestinal-related stimuli
CBT: cognitive behavioral therapy
CPAQ-A8: Chronic Pain Acceptance Questionnaire – Adolescent Short Form
DSQ: daily short questionnaire
FAP-NOS: functional abdominal pain not otherwise specified
FAPD: Functional abdominal pain disorder
FPS-R: Faces Pain Rating Scale – revised
HAPYS: The Health Anxiety by Proxy Scale
HRD: heart rate discrimination
IBS: irritable bowel syndrome
IBS-BRQ: irritable bowel syndrome behavioral response questionnaire
ICBT: internet-delivered cognitive behavioral therapy
ICD-10: International Statistical Classification of Diseases, Tenth Revision
IgA: Immunoglobulin A
PedsQL: Pediatric Quality of Life Inventory
REDCap: Research Electronic Data Capture
SCAS-S: Spence Children Anxiety Scale – Short
SCED: single case experimental design study
SCRIBE: Single-Case Reporting guideline In BEhavioural interventions
SOL: Stop, observe, let go
SPIRIT: Standard Protocol Items Recommendations for Interventional Trials
TSH: thyroid stimulating hormone
VSI: Visceral Sensitivity Index
VSI-C: Visceral Sensitivity Index-Children
